# Management of Leigh syndrome due to *NDUFAF6* variants

**DOI:** 10.1016/j.ymgmr.2019.100456

**Published:** 2019-01-30

**Authors:** Josef Finsterer, Fulvio A. Scorza

**Affiliations:** a Krankenanstalt Rudolfstiftung, Messerli Institute, Veterinary University of Vienna, Vienna, Austria; b Disciplina de Neurociência, Escola Paulista de Medicine/Universidade Federal de São Paulo/, (EPM/UNIFESP), São Paulo, Brazil

**Keywords:** Mitochondrial, Leigh syndrome, Phenotype, Genotype, Epilepsy, Stroke-like episode

We read with interest the article by Baide-Mairena et al. about three siblings with early-onset Leigh syndrome due to compound heterozygous variants in the *NDUFAF6* gene [1]. We have the following comments and concerns.

We do not agree with the statement that thiamine is the only compound effective in Leigh-syndrome [1]. In case Leigh syndrome is due to primary coenzyme-Q deficiency, high dose administration of coenzyme-Q can be highly beneficial [2].

Since the basal ganglia lesions were not only hyperintense on T2/FLAIR sequences but also on diffusion-weighted images (DWI) [1], we should be informed whether apparent diffusion coefficient (ADC) maps showed a corresponding hyperintense or hypointense signal. This is of relevance since ADC hyperintensity would suggest the presence of a stroke-like lesion (SLL), which is the morphological equivalent of a stroke-like episode (SLE). SLEs are potentially treatable by application of NO-precursors, particularly l-arginine [3]. It is also of relevance as Leigh syndrome has been reported to manifest in the cerebrum with SLEs [4]. Since SLLs show up as hyperperfusion in the acute phase on PWI, we should be informed if the basal ganglia lesions revealed an increased perfusion at the stage when DWI signals were hyperintense.

Since Leigh syndrome may manifest clinically with seizures [5], we should be informed if the individual or family history was positive for seizures or epilepsy in the index cases or their first-degree relatives, and if ever paroxysmal activity was recorded on electroencephalography in any of the three presented patients.

Overall, this interesting study could be more meaningful by providing more extensive information about the phenotype of the included siblings, in particular if any of them ever had developed seizures, if the ADC maps were hyper- or hypointense on cerebral MRI, and if the discussion about treatment strategies in patients with Leigh syndrome was revised.

## Conflicts of interest

There are no conflicts of interest.

## Funding

No funding was received.

## Author contribution

JF: design, literature search, discussion, first draft, FAS: critical review, literature search.
